# Assessing the species composition of tropical eels (Anguillidae) in Aceh Waters, Indonesia, with DNA barcoding gene *cox1*.

**DOI:** 10.12688/f1000research.10715.1

**Published:** 2017-03-13

**Authors:** Zainal A. Muchlisin, Agung Setia Batubara, Nur Fadli, Abdullah A. Muhammadar, Afrita Ida Utami, Nurul Farhana, Mohd Nor Siti-Azizah

**Affiliations:** 1 Department of Aquaculture, Faculty of Marine and Fisheries, Syiah Kuala University, Banda Aceh, Indonesia; 2 School of Biological Sciences, Universiti Sains Malaysia, Penang, Malaysia

**Keywords:** Sidat, Ileah, Anguilla bicolor, Anguilla marmorata, DNA barcoding

## Abstract

The objective of the present study was to evaluate the species diversity of eels native to Aceh waters based on genetic data. Sampling was conducted in western coast waters of Aceh Province, Indonesia, from July to August 2016. Genomic DNA was extracted from the samples, a genomic region from the 5’ region of the *cox1* gene was amplified and sequenced, and this was then used to analyse genetic variation. The genetic sequences were blasted into the NCBI database. Based on this analysis there were three valid species of eels that occurred in Aceh waters, namely *Anguilla marmorata*, *A. bicolor bicolor*, and *A. bengalensis bengalensis.*

## Introduction

There are 114 species of freshwater and brackish water fish distributed around 17 sampling locations across Aceh waters ^1^. Several of these have the potential for aquaculture, e.g. the *Anguilla* spp. of tropical eels, locally known as sidat or illeah in Acehnese language ^2– 3^. Based on morphological characteristics, only two species of eels have been recorded in Aceh waters, *Anguilla bicolor* and *Anguilla marmorata* ^1^, but it is believed that the true number of species is greater because some parts of the inland waters in Aceh province have not been explored yet. According to Miller and Tsukamoto ^4^, there are 19 species of eels that have been identified worldwide, 7 of which are found in Indonesian waters ^5^. It is therefore very likely that new species will be found in Aceh waters.

For fisheries management it is crucial to identify these species in order to plan a better conservation strategy, since each one has unique behavioral patterns, and should be independently managed. Eels are very similar morphologically, so it is very difficult to distinguish one species from the other based on morphological characteristics only. Analysing genetic data through DNA barcoding can solve this problem ^6^, so that the true number of eel species living in the waters of Aceh can be evaluated. The objective of the present study was to verify the taxonomic status of eels in Aceh waters by amplifying the *cox1* gene and analysing the genetic data.

## Methods

The study was conducted on the western coast of Aceh Province, Indonesia, from July to November 2016. The samples were processed and analyzed in the School of Biological Sciences, Universiti Sains Malaysia. Sampling was done at night from 18.00 to 06.00 hours. Adult eels were caught using line fishing, while traps were used to catch glass eels. Eel larvae are called glass eels; they have translucent white bodies and measure about 5–10 cm. The length of adult eels is species dependent but most measure between 40–120 cm.

Approximately 1 cm ^2^ of caudal fin tissue was taken from each specimen using a sterile procedure to avoid contamination of specimens. The tissue was placed into 2.0 ml tubes containing 96% alcohol. Genomic DNA was isolated using Aqua Genomic DNA solution following the manufacturer’s protocol ^7– 8^. DNA electrophoresis was carried out on a 0.8% agarose gel at 100V. The quality and quantity of extracted DNA was assessed using a spectrophotometer. A genomic region approximately 655 bp in size was amplified from the 5’ region of the Mitochondrial Cytochrome Oxidase Subunit I ( *cox1*) gene following the protocol from Ward *et al.* ^9^ with these primer pairs:

FishF1: 5’TCAACCAACCACAAAGACATTGGCAC3’

FishR1: 5’TAGACTTCTGGGTGGCCAAAGAATCA3’

After amplification, PCR products were run on 1.2% agarose gels at 100V. The clearest band was selected and purified using purification kits (PCR Clean-Up System, Promega), following the manufacturer's protocol. The purified products were run on 1.2% agarose gels at 100V to check for bands and only clear products were sent for sequencing to First BASE Laboratory Sdn Bhd in Kuala Lumpur, Malaysia. All obtained sequences were edited and aligned using MEGA 6.0 program ^10^. Multiple sequence alignments were performed on the edited sequences with Cluster *W*, which is integrated into the MEGA 6.0 program. The sequences were then blasted into the NCBI database to compare and identify species. Nucleotide divergence among sequences was estimated for their genetic distance by Neighbour-Joining (NJ) based on Kimura 2 parameter. NJ was also used to construct phylogenetic trees to determine genetic relationships among haplotypes.

## Statement on animal ethics

All procedures involving animals were conducted in compliance with The Syiah Kuala University Research and Ethics Guidelines, Section of Animal Care and Use in Research (Ethic Code No: 958/2015). Please refer to Supplementary File 1 for the completed ARRIVE guidelines checklist.

## Results

Genomic DNA from the 5’ region of the *cox1* gene from a total of 13 glass eel samples and 31 adult eel samples were successfully amplified ( Table 1). The results from NCBI BLAST identified two species of eel from adult eel samples, shortfin eel *A. bicolor bicolor* and giant mottled eel *A. marmorata*. In addition, there were three species of eels that were recognized among the glass eel samples, namely *A. bicolor bicolor*, *A. marmorata* and Indian mottled eel *A. bengalensis bengalensis.* A total of 20 haplotypes, consisting of 3 haplotypes of the *A. bengalensis bengalensis*, 1 haplotype of the *A. marmorata*, 15 haplotypes of the *A. bicolor bicolor* and 1 haplotype of the *Uroconger lepturus* (out-group) were produced from 44 samples ( Table 2), out of 132 variable sites. and a haplotype diversity (Hd) of 0.8742. The haplotype number four belongs to *A. marmorata* and was shared by 9 samples from 4 different locations. The haplotype number 5 belongs to *A. bicolor bicolor* and was shared by 13 samples from 6 locations. All of the haplotype sequences have been deposited in the NCBI GenBank with accession numbers KY618767 to KY618795.

**Table 1.** Total sample and code of the tropical eels collected from Aceh waters.

District	Sampling site	Total sample	Stage
Aceh Besar	Beureunut River	13	Glass eels
	Kajhu swamp	5	Adult
	Tibang reservoir	5	Adult
	Aceh River	1	Adult
	Pulo Aceh Island	1	Adult
Nagan Raya	Geutah River	2	Adult
Gayo Lues	Alas River (Blangkejeren)	1	Adult
Aceh Barat Daya	Kuala Batee River	1	Adult
Aceh Singkil	Singkil swamp	5	Adult
Aceh Jaya	Lamno River	1	Adult
Aceh Tenggara	Alas River	4	Adult
Aceh Barat	Meurebo River	3	Adult
Pidie Jaya	Ulim River	1	Adult
Aceh Selatan	Terbangan	1	Adult
Total		44

**Table 2.** Haplotypes according to species and sampling location.

Haplotype	Species	Total sample	Sampling location
1	*Anguilla bengalensis bengalensis*	2	Beureunut River (Glass eels)
2	*Anguilla bengalensis bengalensis*	1	Beureunut River
3	*Anguilla bengalensis bengalensis*	2	Beureunut River
4	*Anguilla marmorata*	9	Beureunut River, Blangkeujeren, Geutah River, Alas River,
5	*Anguilla bicolor bicolor*	13	Beureunut River, Kajhu swamp, Singkil swamp, Meurebo River, Ulim River, Tibang reservoir
6	*Anguilla bicolor bicolor*	1	Beureunut River
7	*Anguilla bicolor bicolor*	1	Beureunut River
8	*Anguilla bicolor bicolor*	1	Beureunut River
9	*Anguilla bicolor bicolor*	3	Aceh River, Pulo Aceh River, Terbangan
10	*Anguilla bicolor bicolor*	1	Kuala Batee River
11	*Anguilla bicolor bicolor*	1	Kajhu swamp
12	*Anguilla bicolor bicolor*	1	Kajhu swamp
13	*Anguilla bicolor bicolor*	1	Meurebo River
14	*Anguilla bicolor bicolor*	1	Meurebo River
15	*Anguilla bicolor bicolor*	1	Lamno River
16	*Anguilla bicolor bicolor*	1	Singkil swamp
17	*Anguilla bicolor bicolor*	1	Tibang reservoir
18	*Anguilla bicolor bicolor*	1	Tibang reservoir
19	*Anguilla bicolor bicolor*	1	Tibang reservoir
20	*Uroconger lepturus* (out-group)	1	Beureunut River
Total	3 species eels (minus out-group)	44	14 locations

Therefore, the study revealed that there are three valid species of tropical eels found in Aceh waters: *A. bicolor*, *A. marmorata,* and *A. bengalensis;* the last species being a newly recorded species in Aceh waters. The study indicates that multiple species of glass eels migrate from the sea into freshwater. One interesting finding was that one sample of conger eels ( *Uroconger lepturus*) was detected among the Tropical glass eel samples. This is indicatory of DNA barcoding being successful in identifying species of eels in Aceh waters which cannot be identified by biometric data. Genetic data has become an important tool in assessing gene flow between marine populations ^11^, species identification ^12^ and monitoring the resources of marine fisheries ^13^.

**Figure 1.**
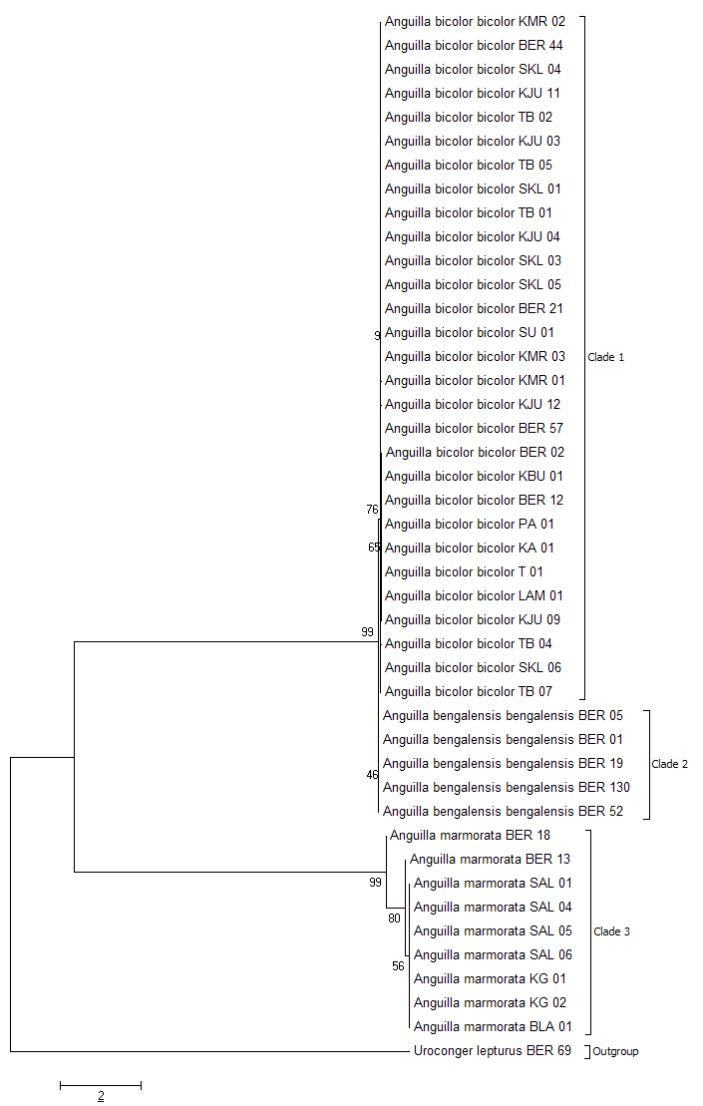
The phylogenetic tree of individual of *Anguilla* samples using the Neighbour-Joining (NJ) method.

The genetic divergence between *A. bicolor* and *A. marmorata* was 5.0%, between *A. bicolor* and *A. bengalensis* it was 6.7% and between *A. marmorata* and *A. bengalensis* genetic divergence was 4.0% ( Table 3). The phylogenetic tree showed a close relationship between *A. marmorata* and *A. bengalensis* ( Figure 1). Based on IUCN ^14^ data, *A. bengalensis bengalensis* and *A. bicolor bicolor* are categorized as near threatened, while the status of *A. marmorata* is on least concern. However, based on direct sampling in Aceh waters the shortfin eels are still abundant and most frequently caught, and are distributed over a wide range of areas including small streams, marshes, peat swamp, estuaries and irrigation channel in paddy fields ^1, 15^. Indian mottled and giant mottled eels on the other hand have been very rarely caught and are generally only found in large rivers directly connected to the sea.

**Table 3.** The genetic distance between three species of Anguilla.

No	Species	1	2	3
1	*Anguilla bicolor bicolor*	-	-	-
2	*Anguilla marmorata*	5.0		-
3	*Anguilla bengalensis* *bengalensis*	6.7	4.0	-

## Conclusion

It is concluded that three species of tropical eels are found in Aceh waters, namely, *A. marmorata, A. bicolor bicolor,* and *A. bengalensis bengalensis* where *A. bengalensis bengalensis* is the newly recorded species.

## Data availability

The data referenced by this article are under copyright with the following copyright statement: Copyright: © 2017 Muchlisin ZA et al.

Data associated with the article are available under the terms of the Creative Commons Zero "No rights reserved" data waiver (CC0 1.0 Public domain dedication). 

Sequenced DNA of Tropical eels from Aceh waters can be found in the NCBI GenBank repository ( https://www.ncbi.nlm.nih.gov/genbank/) with accession numbers KY618767 to KY618795.
