# Supplementary material for: Assessing the species composition of tropical eels (Anguillidae) in Aceh Waters, Indonesia, with DNA barcoding gene cox1
Source: F1000Res. 2017 Mar 13;6:258. [Version 1] doi: 10.12688/f1000research.10715.1 (PMC5428524; doi:10.12688/f1000research.10715.1)
Supplement: Peer review history

# Peer review: Referee response for version 1

**DOI:** 10.5256/f1000research.11554.r22410

**Published:** 2017-05-11

**Authors:** Murugaiyan Kalaiselvam

**Affiliations:** 1 Centre of Advanced Study in Marine Biology, Faculty of Marine Sciences, Annamalai University, Tamil Nadu, India

1. In Introduction, add few more points regarding the importance of Genetic identification, and demerits of conventional identification strategies (Only one point had been given in introduction for name sake, add few more)
2. In addition, author will add the possible outcome after identification of eels, in what way this work serves to the research community?
3. Prior to molecular identification, the author done the sample identification by morphometric characters? Though it’s a old procedure, it is of much importance and the results of morphometric analysis acts as an base step for identification.
4. Data on Taxonomic characters will serve as a guide for identification of the same, whereas having sequences on hands will not be useful for further reference.
5. Authors stated that only two species of eels have been recorded in Aceh waters and in results they recorded 3 species with molecular results? Thus the morphometric identification of eels should be included so that what are the distinct features of 3 eels can be clarified to the readers.
6. Generally for DNA barcoding analysis lateral tissue from the left side of fish will be taken in to consideration, but the authors had chosen caudal fin tissue, is there any justification for taking the caudal fin tissue, if so justify that and add proper reference for that methodology.  
7. Totally 13 glass eel and 31 adult eels were, so totally out of the 44 samples, the results inferred belongs to only 3 species, so care should be taken prior to analyzing the samples for molecular identification as it is cost effective process and wastage of chemicals.  
8. How did author arrived the genetic divergence?
9. Materials needs clear cut procedures and reference alone doesn’t enough:
  - Genomic DNA was isolated using Aqua Genomic DNA solution following the manufacturer’s protocol ^7-8^
  - Mitochondrial Cytochrome Oxidase Subunit I ( *cox1*) gene following the protocol from Ward *et al. ^9^*
10. Discussion part need to be written with the comparative studies made by author authors regarding the availability of eel in study area, identification problems, and results of the present study with respect to molecular identification and highlights the importance of the obtained results.
11. Conclusion seems to be the result and what is the inference made from the study should be written precisely and accurately.

I have read this submission. I believe that I have an appropriate level of expertise to confirm that it is of an acceptable scientific standard, however I have significant reservations, as outlined above.

# Peer review: Referee response for version 1

**DOI:** 10.5256/f1000research.11554.r21916

**Published:** 2017-04-18

**Authors:** Mudjekeewis D. Santos

**Affiliations:** 1 Genetic Fingerprinting Laboratory, National Fisheries Research and Development Institute, Quezon City, Philippines

This paper has some scientific merit in providing new information about species composition of eel in Aceh waters and in using COX1 gene as a marker. As such I find it suitable for indexing after major revisions:

1. Authors need to highlight in the Introduction the existing information/status about eels in Aceh waters.
2. In addition, they need to relate the study on existing eel trade (domestic or export) in the area if any since this is the main threat for the said species.
3. The paper of Asis et al. (2014) ^1^ would help enrich the objective of this paper.
4. The reference DNA sequences used in the paper/trees are not clear. Did the authors established their own reference sequences for cox1? This should be indicated or made clear.

I have read this submission. I believe that I have an appropriate level of expertise to confirm that it is of an acceptable scientific standard, however I have significant reservations, as outlined above.

## References

[1] AsisAMLacsamanaJKSantosMD: Illegal trade of regulated and protected aquatic species in the Philippines detected by DNA barcoding. *Mitochondrial DNA A DNA Mapp Seq Anal*.2016;27(1) : 10.3109/19401736.2014.913138 659-66 10.3109/19401736.2014.913138 24841434

# Peer review: Referee response for version 1

**DOI:** 10.5256/f1000research.11554.r20933

**Published:** 2017-03-22

**Authors:** Salman Abdo Al-Shami

**Affiliations:** 1 Department of Biology, University College of Taymma, University of Tabuk, Tabuk, Saudi Arabia

The manuscript in hands present interesting information about species diversity of tropical eels (Anguilla spp.) in Aceh, Indonesia. Although the manuscript is nicely presented, few justifications and clarification are still required. 

**Title **

- For general readers, using the word "waters" may make the readers confused about what type of water bodies in which the samples collected from. For example, how about using "coastal line" instead of "waters" or just delete the word "waters".
- Please replace the family name of eels with "Anguilla spp." to be more precise. 

**Abstract**

 I believe adding an introductory sentence will make the research summary more meaningful. This introductory sentence will highlight the importance of the study and make a sound justification of the study objectives. 

- Should be read "...the present study is to evaluate..."
- Should be read "the western"
- "coastal waters" change into "coastal line" or "marine environment".
- Add semicolon after "namely;"
- Add comma after "Based on this analysis"
- The word "genomic" makes me confused. Is it mitochondrial or genomic gene? Please correct me if I am wrong.

**Introduction**

- I would suggest extending the introduction in a way that gives the readers a comprehensive background about the research based on the available literature.
- It would be nice to start the introduction with an introductory paragraph to give the readers the brief understanding about the research context.

**Methods**

 The procedures and tools used to collect the eel samples should be described elaborately. For example, it was stated that traps were used to catch the glass eels. It was not mentioned what type of traps? How did the researchers set the trap? For how long did they leave the traps?

- It will be excellent if the authors provide a geographical map showing the approximate locations of sampling sites.
- It would be nice to add a reference to the method of sampling the eels' tissue.
- Please add a reference to "Kimura 2 parameters"

**Results**

 The presentation of the results is adequate and no further corrections or additions are required.

- Figure 1: please add "Anguilla spp." to the figure caption.

I have read this submission. I believe that I have an appropriate level of expertise to confirm that it is of an acceptable scientific standard.
